# The crosstalk between the caspase family and the cGAS‒STING signaling pathway

**DOI:** 10.1093/jmcb/mjab071

**Published:** 2021-10-27

**Authors:** Yongai Xiong, Yan-Dong Tang, Chunfu Zheng

**Affiliations:** 1 Key Laboratory of Basic Pharmacology of Guizhou Province and School of Pharmacy; Key Laboratory of Basic Pharmacology of Ministry of Education and Joint International Research Laboratory of Ethnomedicine of Ministry of Education, Zunyi Medical University, Zunyi, China; 2 Department of Immunology, School of Basic Medical Sciences, Fujian Medical University, Fuzhou, China; 3 State Key Laboratory of Veterinary Biotechnology, Harbin Veterinary Research Institute of Chinese Academy of Agricultural Sciences, Harbin, China; 4 Department of Microbiology, Immunology and Infectious Diseases, University of Calgary, Calgary, Alberta, Canada

**Keywords:** cGAS‒STING, caspases, innate immunity, signaling pathway

## Abstract

Edited by Jiarui Wu

Cytosolic nucleic acid sensors are critical for sensing nucleic acids and initiating innate immunity during microbial infections and/or cell death. Over the last decade, several key studies have characterized the conserved mechanism of cyclic guanosine monophosphate‒adenosine monophosphate synthase (cGAS) and the downstream signaling adaptor stimulator of interferon genes (STING) initiating the innate immune signaling pathways. Aside from its primary involvement in microbial infections and inflammatory diseases, there is growing interest in the alternate roles of cGAS‒STING-mediated signaling. Caspase family members are powerful functional proteins that respond to cellular stress, including cell death signals, inflammation, and innate immunity. Recent studies have uncovered how the caspase family cooperates with the cGAS‒STING signaling pathway. Most caspase family members negatively regulate the cGAS‒STING signaling pathway. In turn, some caspase family members can also be modulated by cGAS‒STING. This review gives a detailed account of the interplay between the caspase family and the cGAS‒STING signaling pathway, which will shed light on developing novel therapeutics targeting the caspase family and cGAS‒STING signaling in antiviral innate immunity, cancer, inflammatory, and autoimmunity.

## Introduction

Apoptosis, also known as the noninflammatory type of programmed cell death, is a consequence of irreversible changes in cells caused by pro-apoptotic stimulating factors such as endoplasmic reticulum (ER) stress and reactive oxygen, which activate the caspase family members (D'Arcy, 2019; Xu et al., 2019; Obeng, 2021), to maintain the body homeostasis. It is usually characterized by intracellular DNA degradation, pyknosis and fragmentation of cell nucleus, and apoptotic body formation. Accumulating evidence has shown that apoptosis plays a vital role in human embryogenesis, the perfection of the nervous system, the regulation of immune function, and the incidence and development of diseases (Kawamoto et al., 2016; Garg and Agostinis, 2017; Van Opdenbosch and Lamkanfi, 2019).

The initiation of apoptosis hinges on activating a series of caspases (Man and Kanneganti, 2016; Bolívar et al., 2019). Caspases are cytoplasmic cysteine proteases with similar structural properties. Most caspases have similar structures with a cysteine residue in the active site and can specifically cleave the peptide bond on the target protein’s aspartic acid (D) residue (Julien and Wells, 2017). Caspases are closely related to eukaryotic cell apoptosis and involved in multiple cellular processes, such as programmed cell death, inflammasome activation, and differentiation (Seo and Rhee, 2018; Kesavardhana et al., 2020). Caspases are classified into two types based on their functions: inflammatory caspases and apoptotic caspases (Nagata, 2018). Inflammatory caspases include caspase-1, caspase-4, caspase-5, caspase-11, caspase-12, and caspase-13. Apoptotic caspases are mainly divided into apoptosis initiating caspases and apoptosis executing caspases. Initiating caspases include caspase-2, caspase-8, caspase-9, and caspase-10 and executing caspases include caspase-3, caspase-6, caspase-7, and caspase-14. Caspases constitute a protease cascade that can rapidly amplify cell death and inflammation responses. Recently, caspase family members have been demonstrated to modulate innate immunity via affecting pattern recognition receptors (PRRs) on the plasma membrane (McIlwain et al., 2013; Chen et al., 2017).

The cyclic guanosine monophosphate‒adenosine monophosphate (cGAMP) synthase (cGAS)‒stimulator of interferon genes (STING) signaling pathway plays a vital role in the innate immune system, which is the first-line defense against pathogen invasion (Motwani et al., 2019; Zhang et al., 2020). The cGAS‒STING signaling pathway mediates the innate immune response to restrict infections caused by various pathogenic microorganisms containing DNA and detects tumor-derived DNA to induce antitumor immune responses. Numerous studies over the last decade have shown that the cGAS‒STING signaling pathway plays an important regulatory role in pathogen infection, malignancies, and autoimmune disorders by regulating both innate immunity and adaptive immunity (Cheng et al., 2020; Kwon and Bakhoum, 2020; Wan et al., 2020). The cGAS‒STING signaling pathway can help the body to resist viral infection and tumor cells; however, it can also induce severe inflammation and affect autoimmunity when excessively activated (Hopfner and Hornung, 2020). Understanding the molecular biology and regulatory mechanism of the cGAS‒STING signaling pathway is critical to balance immunity and inflammation and to explore novel targets for inflammatory disorders.

The crosstalk between caspases and the cGAS‒STING signaling pathway has recently received much attention. Numerous studies have shown that caspases have a critical role in regulating innate immune responses by mediating the cGAS‒STING pathway. The molecular mechanisms of caspases’ interaction with the cGAS‒STING signaling pathway are reviewed here. We are particularly interested in how the caspase family regulates the cGAS‒STING signaling pathway and how it is involved in innate immune responses, either directly or indirectly. Meanwhile, we also focus on the modulation of cGAS‒STING signaling on the caspase family. The comprehensive elucidation of the interplay between the caspase family and the cGAS‒STING signaling pathway will be useful in identifying novel therapeutic targets and potential therapies against infectious diseases and cancer (Chen et al., 2017).

### The regulatory roles of caspases in the cGAS‒STING signaling pathway

cGAS is an intracellular PRR (Thomsen et al., 2020). cGAS recognizes viral, bacterial, protozoal, mitochondrial, and self-DNA from the tumor or dead cells in the cytosol to activate downstream signaling pathways. After sensing DNA, the activated cGAS can synthesize cGAMP from ATP and GTP. cGAMP can directly activate STING on the ER. After activation, STING is translocated from the ER to the Golgi apparatus and recruits and activates the downstream adaptor TANK-binding kinase 1 (TBK1), promoting the nuclear importation of interferon regulatory factor-3 (IRF3) and NF-κB, resulting in the transcription of interferon-related genes, type I interferon (IFN-I), and inflammatory factors, thereby enhancing immune responses. The cGAS‒STING pathway has been proved to play an important role in regulating cell metabolism, autophagy, cell death, intestinal inflammation, nonalcoholic fatty liver, kidney fibrosis, etc. (Decout et al., 2021). Except for the involvement in the intrinsic apoptotic signaling pathway, the caspase family has been demonstrated to regulate IFN-I production by modulating cGAS‒STING signaling.

### cGAS

cGAS is a nucleotidyltransferase with a nonstructural and nonconserved N-terminus of 130‒150 residues and a highly conserved Mab21 domain containing a zinc-binding dimerization motif. cGAS is activated by DNA-dependent dimerization and multimerization. The less conserved N-terminus of cGAS has sequence-independent DNA-binding activity to promote cGAS activation by DNA, whereas the C-terminal catalytic domain owns three DNA-binding sites, named ‘site A’, ‘site B’, and ‘site C’, which cooperatively bind to dsDNA and contribute to cGAS activation (Margolis et al., 2017; de Oliveira Mann and Hopfner, 2021).

Caspase-1 is a critical protein in the inflammasome, as it can cleave protein precursors to produce active interleukin-1 (IL-1) and IL-18, thereby contributing to the inflammatory responses (Sun and Scott, 2016). Caspase-1 inhibits interferon-β (IFN-β) production via cleaving cGAS and dampening cGAS‒TBK1‒IRF3 signaling in *Mycobacterium bovis*-infected cells (Liao et al., 2021). In *M. bovis*-infected bone marrow-derived macrophages, caspase-1 cleaves cGAS, resulting in reduced TBK1 phosphorylation and IRF3 nuclear translocalization, eventually reducing IFN-β production. Infection with *M. bovis* induces cytokine production via PRR-mediated signaling pathways, including IFN-I and IL-1β. Excessive IFN-I impairs host resistance to *M. bovis* infection. As a result, accurate control over IFN-I production is beneficial in reducing pathogenic damage and bacterial burden. Viral infection activates innate immune responses, produces various cytokines including IFN-I, and activates inflammasome responses and programmed cell death. Tight regulation of inflammatory cytokine is critical for efficient immune responses to resolve the infection without initiating host pathology. Accordingly, precise regulation of inflammasomes and IFN-I production during infection should be investigated, and efforts should be made to avoid inadequate or excessive innate immune responses. Wang et al. (2017) demonstrated that caspase-1 cleaved cGAS and inhibited IFN-I production mediated by cGAS‒STING in the casp1^–/–^ macrophages, and caspase-1 deficiency enhanced IFN-I production during DNA virus infections. The underlying mechanism revealed that upon canonical and noncanonical inflammasome activation, caspase-1 targets and suppresses the cGAS‒STING signaling pathway by direct binding to and cleaving cGAS at D^140/157^, resulting in reduced cGAMP levels and inhibition of cGAS‒STING-mediated IFN-I production (Wang et al., 2017).

The cGAS‒STING pathway is activated during DNA virus infection via DNA sensor cGAS sensing cytosolic dsDNA from herpes simplex virus-1 (HSV-1), Kaposi's sarcoma-associated herpesvirus (KSHV), valacyclovir, adenovirus, and hepatitis B virus. HSV-1 successfully establishes acute and latent infections in humans by counteracting host antiviral innate immune responses and has evolved various strategies to evade host antiviral innate immunity and cellular survival-associated pathways (Zhu and Zheng, 2020). HSV-1 activates caspase-1 and reduces IFN-β release, suggesting that caspase-1 is involved in the negative regulation of IFN-I production (Danastas et al., 2020). Furthermore, it was shown that caspase-1-deficient cells have a higher level of activation of the cGAS‒STING signaling pathway (Heidegger et al., 2017). Zheng et al. (2018) showed that Zika virus (ZIKV) activates the NS1‒caspase-1 axis and induces inflammation to abrogate antiviral innate immunity. ZIKV activates host inflammasome responses to stabilize caspase-1, and caspase-1 cleaves cGAS, dampening the cGAS‒STING-mediated antiviral innate immune responses and enhancing ZIKV replication (Zheng et al., 2018). The antagonistic mechanism employed by ZIKV to manipulate the interplay between inflammasome and IFN-I revealed that ZIKV has evolved a strategy to counter IFN-I’s antiviral responses and evade host antiviral innate immunity.

Caspases-3, like caspase-1, adversely regulates the cGAS‒STING signaling pathway. Ning et al. (2019) discovered that the activated caspase-3 cleaves cGAS, mitochondrial antiviral signaling (MAVS) and IRF3 in DNA or RNA virus-infected cells, resulting in restrained IFN-I production (Ning et al., 2019). Furthermore, caspase-3 cleaves cGAS at D^319^ and IRF3 at D^121/125^, thus keeping apoptotic cells immunologically silent and negatively regulating DNA virus-induced cytokine production. It also cleaves MAVS at D^429/490^ and IRF3, leading to restrained interferon production during an RNA virus infection. As a result, caspase-3 regulates innate immune activation during viral infection and/or apoptotic activation. Interestingly, viral infection has been demonstrated to induce cell apoptosis, and the activation of apoptotic caspases can cleave cGAS, MAVS, and IRF3, which may be an important mechanism for viruses to evade immune surveillance.

Caspases also reduce the immunogenicity of radiation (Buqué et al., 2019). Caspase-3-incompetent TSA cells secrete more IFN-I than their control counterparts upon exposure to radiation (Vanpouille-Box et al., 2017), suggesting that caspase-3 limits the accumulation of permeabilized mitochondria, and hence the cells are prone to secrete IFN-I downstream of the mitochondrial DNA (mtDNA)-driven activation of cGAS. Furthermore, caspase-3 accelerates the functional inactivation and structural breakdown of dying cells, and the terminal inactivation of dying cells limits their immunogenicity in response to radiation therapy due to decreased IFN-I production (Rodriguez-Ruiz et al., 2019). Thus, caspase inhibitors stand out as prospective combinatorial partners for improving the immunogenicity of radiation therapy in the clinic.

Nonetheless, as evidenced by the absence of T-cell proliferation in the presence of caspase-8 mutations, caspase activation is required for human T-cell function (Chun et al., 2002). Consequently, it is conceivable that using a pan-caspase inhibitor might promote radiation therapy-induced IFN-I and hinder T-cell responses by inactivating caspase-8. During noncanonical inflammasome activation induced by lipopolysaccharide (LPS), cGAS may also be cleaved by caspase-4, caspase-5, and caspase-11 in a way different from caspase-1 (Wang et al., 2017). Caspase-4, caspase-5, and caspase-11 have been shown to interact with and activate caspase-1 (Fang and Peng, 2021). Furthermore, caspase-4, caspase-5, and caspase-11 activate the NLRP3 inflammasome in response to Gram-negative bacteria and intracellularly delivered LPS, placing caspase-4, caspase-5, and caspase-11 as activators of caspase-1 to promote caspase-1-dependent cleavage of IL-1β and IL-18 (Kayagaki et al., 2011; Shi et al., 2014). Even though caspase-4 and caspase-5 in humans and caspase-11 in mice are capable of cGAS cleavage in LPS-induced noncanonical inflammasome activation, the specific target sites need further investigation.

Based on these findings, caspase-1, caspase-3, caspase-4, caspase-5, and caspase-11 can cleave cGAS, limiting cGAS-mediated IFN-I production during DNA virus or bacteria infection. The apoptotic caspase suppression enhances IFN-I production in virus-infected cells, suggesting that these caspases play a negative role in guaranteeing proper innate immune activation in response to pathogenic infection. However, caspase-mediated cleavage and inactivation of cGAS probably prevent IFN-I-mediated autoimmunity, although this may come at the expense of antiviral immunity. These concerns must be taken into account in the development of future caspase-targeting drugs.

### STING/TBK1

STING, also known as transmembrane protein 173 and MPYS/MITA/ERIS, is a protein that in humans is encoded by the STING1 gene. STING is often regarded in mammalian cells as a factor dedicated to defense innate immunity triggered by DNA viruses (Zhu et al., 2019). STING plays an important role in cancer, immunity, and inflammation (Kumar, 2019). Mitochondria are cellular organelles that regulate various biological activities, including energy generation, metabolism, cell death, and inflammation. Mitochondria contain a powerful innate immunity agonist: their own mtDNA despite this seemingly symbiotic interaction. The release of mtDNA into the cytoplasm and the extracellular milieu activates many PRRs and their downstream innate immune responses, including cGAS‒STING, TLR9, and inflammasome formation, which potentiates IFN-I production and confers broad viral resistance. Mitochondrial apoptosis mediated by BAX and BCL2 may result in mtDNA leaking into the cytoplasm, activating the cGAS‒STING signaling pathway in caspase-9-deficient cells (Rongvaux et al., 2014). In addition, pharmacological inhibition or gene knockout of caspase-3, caspase-7, and caspase-9 induces dying cells to secrete IFN-β and blocks STING recruitment, TBK1 phosphorylation, and IRF3 phosphorylation and dimerization, suggesting that caspase-3, caspase-7, and caspase-9 are negative regulators of mtDNA-induced damage-associated molecular pattern signaling and could downregulate cGAS/STING/TBK1/IRF3-mediated IFN-α/β production (White et al., 2014). Mitochondria and caspases are important in a cell’s decision to survive or die, as well as whether to die inflammatory or immunologically quiet. However, the specific target of these caspases after apoptotic cell death to guarantee immunological silence is unclear.

Furthermore, a caspase-8-like protein (BmCasp8L) from the silkworm (Bombyx mori) was identified as a BmSTING interactor and an inhibitor of BmSTING-mediated BmRelish activation in insect antiviral innate immunity (Hua et al., 2018). Caspase-8 has a role in the prevention of inflammation by inhibiting the activity of inflammasomes (Kang et al., 2013) and IFN-I responses (Rajput et al., 2011).

### IRFs

The IRFs are key transcription factors that play important roles in the interferon signaling pathway and cellular immunity (Mogensen, 2019). IRFs have recently been shown to interact with caspases. Several caspases have been reported to either directly or indirectly regulate IRFs. Caspases, in turn, can be controlled by IRFs.

IRF3 plays a central role in the transcription of cellular antiviral genes, including the INF-β gene. IRF3 modulates the strength and duration of the innate immune responses and is finally targeted for proteasome-mediated degradation. Kovalenko et al. (2009) reported that deficiency of caspase-8 in mouse keratinocytes leads to the hyperactivation of IRF3. The direct cleavage of IRF3 by caspase-8 downregulates the double-stranded RNA-dependent signaling pathway and induction of its downstream interferon-stimulated genes, the major components of the antiviral mediators (Sears et al., 2011). Another study showed that caspase-8 cleaves the polyubiquitinated RIPK1, releasing a RIPK1 fragment that inactivates IRF3 and inhibits RIG-I/MDA-5-mediated antiviral IFN-I production. Caspase-8 deficiency in fibroblastoid cells or hepatocytes enhances IRF3 activation during RNA virus infection (Rajput et al., 2011; Kang et al., 2013). Caspase-8 has also been shown to prevent inflammasome activity and interferon response. Furthermore, RIG-I-mediated signaling activates caspase-8, which initiates a critical intermediary phase in the ubiquitination/proteasome-dependent degradation of IRF3, regulating the dsRNA-dependent signaling pathway (Sears et al., 2011).

In addition, mice lacking caspase-8 in keratinocytes develop cutaneous inflammation, which is not caused by TNF, IL-1, or TLR signaling but rather by an increase in the transcription factors IRF3 and TBK1 in the epidermis. Meanwhile, keratinocytes lacking caspase-8 induce higher amounts of IFN-β and interferon-inducible proteins following transfection of double-stranded DNA than wild-type keratinocytes (Kovalenko et al., 2009). Increased expression of IRF3 and IRF7 has also been reported in BMDCs lacking caspase-8.

Caspase-3 has also been excavated to regulate IRFs, although further investigation is needed. During poly(I:C) transfection, Mutocheluh et al. (2011) showed that caspase-3 indirectly suppresses IRF3 in KSHV-infected cells and participates in normal IRF3 turnover in the absence of vIRF-2, a viral IRF (vIRF) protein of KSHV, which is homologous with IRFs and is an inhibitor of IFN-I production. Alhetheel et al. (2020) found that STAT1 and IRF1 are downregulated in hepatitis C virus-infected individuals’ peripheral blood mononuclear cells, whereas caspase-3 increases (Alhetheel et al., 2020). Although it is a wonderful idea that has to be validated, the involvement of caspase-3 in regulating IRFs appears to be affirmative. However, it is still worth investigating whether it directly targets IRFs as well as the underlying mechanism.

The coronavirus disease 2019 (COVID-19) epidemic is still spreading throughout the world. Evidence suggests that in severe COVID-19 patients, the protective IFN-I response is significantly decreased or delayed (Blanco-Melo et al., 2020; Galani et al., 2021). During severe acute respiratory syndrome coronavirus (SARS-CoV) infection, the timing of the interferon response is critical, because delayed IFN-I causes fatal pneumonia (Lee et al., 2020). A delayed IFN-I response has been shown to obstruct viral clearance and cause paradoxical hyper inflammation, worsening the immunopathological response (Channappanavar et al., 2016, 2019; Yang et al., 2021).

SARS-CoV and SARS-CoV-2 infections, interestingly, activate caspase-8, which initiates a signaling cascade. Caspase-8 initiates apoptosis by activating caspase-3. ORF3a from SARS-CoV-2 promotes apoptosis in HEK293T, HepG2, and Vero E6 cells by cleaving caspase-8 and caspase-9 (Ren et al., 2020). Furthermore, apoptosis was observed in SARS-CoV-2-infected cells, as evidenced by the cleavage of apoptotic caspases, caspase-3, caspase-7, caspase-8, and caspase-9 (Karki et al., 2021).

Considering the interactions among caspases, apoptosis, and cGAS‒STING signaling, it is very likely that the delayed IFN-I response of COVID-19 patients is related to apoptosis induced by SARS-CoV-2. Caspase-mediated cleavage of cGAS‒STING prevents IFN-I production, which may be an important cause of the cytokine storm. Hence, the inhibition of apoptosis is conducive to innate immunity to clear SARS-CoV-2 and reduce viral lung injury.

### The modulation of cGAS‒STING signaling on the caspase family

DNA-sensing receptors have recently been shown to cause not just IFN-I production but also apoptosis. The cGAS‒STING axis can trigger a cell death response to detect cytosolic DNA (Paludan et al., 2019). By transcriptionally activating apoptotic regulators and IRF3, the cGAS‒STING signaling pathway can induce apoptosis. The STING system promotes cell death by increasing the production of the pro-apoptotic protein BAX, which mediates mitochondrial outer membrane permeability and caspase-3 activation mediated by caspase-9 (Li et al., 2019a). The degradation of the X-linked inhibitor of apoptosis (XIAP) by IRF3 provides a possible connection to virus-induced apoptosis. Caspases-3, caspase-7, and caspase-9-dependent apoptotic signals are all inhibited by XIAP. Infection with Sendai and vesicular stomatitis virus causes phosphorylation of XIAP at serine 430 by TBK1/IKK *in vivo*, resulting in auto-ubiquitination-mediated proteasomal destruction of XIAP (Nakhaei et al., 2012).

The cytoplasmic cGAS has DNA-binding ability, and when it binds, it generates cGAMP, which activates the STING protein. As a result, STING activates IRF3 and causes intrinsic apoptosis (Wu et al., 2019), a negative feedback regulation mechanism for cells to avoid excessive immunity. HSV-1 infection was reported to induce cGAS-dependent apoptosis in the murine brain. Microglia primarily express IFN-I, which has antiviral action, when the local viral load is low. When the viral burden is substantial; however, cGAS‒STING signaling flips to promote apoptosis by increasing Puma mRNA expression, mitochondrial cytochrome c release, and caspase-3 cleavage, lowering the IFN-I response and perhaps limiting immunopathology. Moreover, both of the cGAS^–/–^ mice and STING^–/–^ mice exhibit impaired induction of caspase-3 cleavage in response to HSV-1 infection, indicating that virus-induced apoptosis occurs via the cGAS‒STING pathway. Thus, the cGAS‒STING pathway exerts not only paracrine activity via IFN-I but also cell-autonomous negative regulation of immune cells via activation-induced apoptosis (Reinert et al., 2021).

In human cell culture and mouse models, a noncanonical inflammasome pathway activates caspase-4 (caspase-11 in mice) and needs cGAS-dependent IFN-β production, and gasdermin D-dependent IL-18 secretion is responsible for retinal pigment epithelium (RPE) degeneration. The cytosolic escape of mitochondrial DNA, which activates cGAS, is triggered by a reduction in DICER1 levels or an accumulation of *Alu* RNA. Furthermore, levels of caspase-4, gasdermin D, IFN-β, and cGAS are increased in the RPE of human eyes with geographic atrophy, indicating that cGAS-driven interferon signaling is a route for mitochondrial damage-induced inflammasome activation (Kerur et al., 2018).

Some immune cells’ apoptosis can also be triggered by the cGAS‒STING pathway. For instance, the activation of STING has been shown to induce T-cell apoptosis. The emerging evidence suggests that STING-induced ER stress has an important influence on the survival of T cells, which has been proved to be related to the regulation of STING on calcium homeostasis and ER stress, as STING gain-of-function mutant disrupts calcium homeostasis causing ER stress that induces T-cell apoptosis (Wu et al., 2019). Also, the ER stress mediated by STING activation primes apoptosis in normal and malignant B cells (Tang et al., 2016).

Collectively, the cGAS‒STING signaling pathway can, in turn, regulate caspase-mediated apoptosis, during which IRF3 plays a core regulatory role. Most of the caspase promotion mechanisms lead to apoptosis culminate in IRF3 activation, highlighting the significance of IRF3 in the virus or inflammation-induced apoptosis. However, the underlying mechanisms by which cGAS‒STING triggers cell death are only partially understood. The exact mechanism of cGAS‒STING regulating the caspase family-mediated cell apoptosis is to be further investigated.

## Conclusion and future perspectives

Caspases are a family of cysteine proteases whose functions have recently received much attention. Some caspases have been shown to engage in the innate immune signaling system in addition to their known involvement in programmed cell death and inflammatory response. Caspases cleave their target proteins through most of their activity, and the substrate specificity governs their cellular function, whether it is maintaining natural immunological homeostasis or triggering autoimmune disorders (Seo and Rhee, 2018). This review briefly introduced the regulation of the cGAS‒STING signaling pathway by apoptotic caspases and highlighted the caspase family’s roles in maintaining immunological silence or promoting inflammatory responses to eliminate invading pathogens.

The cGAS‒STING signaling pathway has a complicated function in the development of infectious diseases and cancer. The activation of cGAS and STING may impact viral infection and cancer development positively or negatively. The cGAS‒STING signaling pathway has gained multiple functions against pathogens and cancer, but the excessive activation of the cGAS‒STING signaling pathway may lead to serious inflammation, autoimmunity, and various diseases. Furthermore, the cGAS‒STING signaling pathway has become an essential mechanism for many viruses and cancer cells to evade detection by immune systems (Saeed et al., 2020). The regulation of the cGAS‒STING signaling pathway by apoptotic caspases plays an important role in innate immune responses.

**Figure 1:**
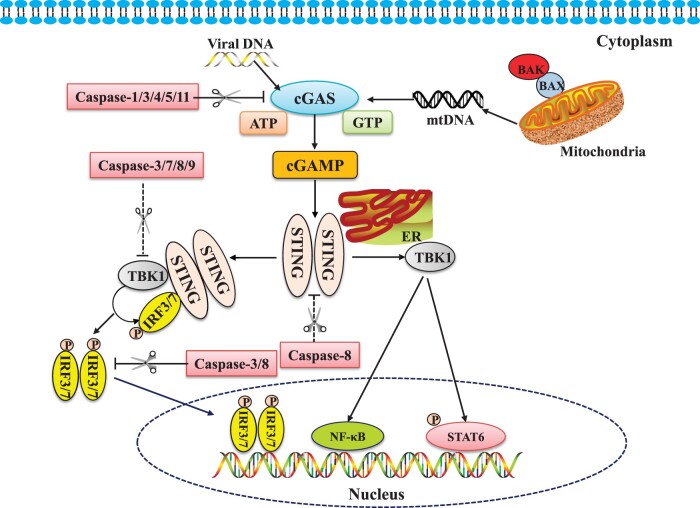
Caspases regulate cGAS‒STING signaling pathways. Caspases can modulate multiple adaptors in the cGAS‒STING signaling pathway. The apoptotic caspase-1, caspase-3, caspase-4, caspase-5, and caspase-11 can directly cleave cGAS and limit cGAS-mediated IFN-I production during DNA virus or bacteria infection. The apoptotic caspase-3, caspase-7, caspase-8, and caspase-9 can inhibit cGAS/STING/TBK1/IRF3-mediated IFN-α/β production, while caspase-8 can also cleave STING to control innate immunity. Furthermore, caspase-3 and caspase-8 negatively regulate the cGAS‒STING pathway resulting in cleavage of IRF3 and IRF7. The pink shield indicates the downregulation of the signaling pathways by caspases.

Most caspase family members negatively regulate the key adaptors of the cGAS‒STING signaling pathway, resulting in suppression of innate immunity. Caspase-1, caspase-3, caspase-4, caspase-5, and caspase-11, e.g. can cleave cGAS, reducing cGAS-mediated IFN-I production after DNA virus or bacteria infection. Caspases-3, caspase-7, and caspase-9 inhibit IFN-I induction by acting on TBK1. Caspase-3 and caspase-8 inhibit IFN-I production by cleaving IRF3 and IRF7. Caspase-8 is also capable of cleaving STING. These findings suggest that the caspase family plays an important regulatory function in innate immunity. The inhibition of the caspase family on the cGAS‒STING signaling pathway was summarized in Figure 1, and the reported caspases’ cleavage sites for that cGAS‒STING signaling pathway were listed in Table 1.

**Table 1:** Sites of caspase cleavage of the cGAS‒STING signaling pathway.

Cleavage site	cGAS	STING	TBK1	IRF3	References
Caspase-1	D^140/157^				Wang et al. (2017)
Caspase-3	D^139^		+	D^121/125^	Ning et al. (2019)
Caspase-4	+				Wang et al. (2017)
Caspase-5	+				Wang et al. (2017)
Caspase-7			+		White et al. (2014)
Caspase-8		+	+	+	Hua et al. (2018); Kovalenko et al. (2009)
Caspase-9			+		White et al. (2014); Rongvaux et al. (2014)
Caspase-11	+				Wang et al. (2017)

However, negative regulation of caspases on the cGAS‒STING signaling pathway is beneficial for autoimmune disorders such as rheumatoid arthritis and systemic lupus erythematosus (Li et al., 2019b) via suppressing the excessive immune amplification and reducing the damage to the body. Meanwhile, it is reminded that when using various caspase inhibitors clinically, it is necessary to fully consider that the improper immune activation may occur when the caspase is inhibited; on the other hand, caspase inhibitors may be used as a new type of immune enhancer.

Furthermore, not all caspases can perform the cleavage of cGAS‒STING, and the importance of caspases in the control of apoptosis, inflammation, cell cycle, and cell differentiation signaling might be increased with a better knowledge of the physiological roles of caspase-2, caspase-6, caspase-10, and caspase-14.

**Figure 2:**
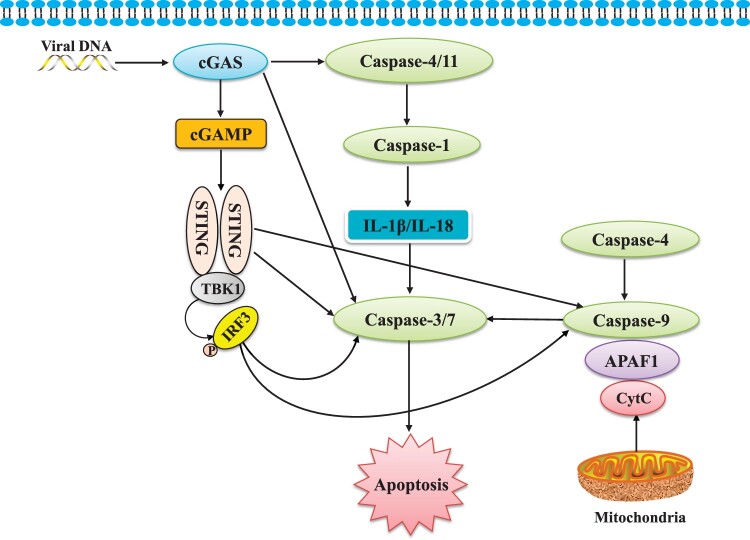
The modulation of cGAS‒STING signaling on the caspase family. The cGAS‒STING signaling pathway modulates the caspase family in terms of apoptosis directly and indirectly. Caspase-4 and caspase-11 can be activated by cGAS. Activation of caspase-4 and caspase-11 leads to caspase-1 activation, which accomplishes the processing of IL-1β and IL-18. IL-1β and IL-18 stimulate caspase-3 and caspase-7 to initiate apoptosis. cGAS can also directly affect caspase-3 and caspase-7 to induce apoptosis. Both STING and IRF3 stimulate the activation of caspase-9 and downstream executioners caspase-3 and caspase-7 to initiate apoptosis.

According to current research, caspase-mediated apoptosis can be induced by cGAS‒STING as an innate immunological response to cancer or infection. Apoptosis, pyroptosis, and necroptosis are all induced by RNA and DNA sensors (Maelfait et al., 2020). These pathways contribute to the production of cytokines and inflammatory mediators and prevent pathogen replication by removing contaminated cells (Jorgensen et al., 2017; Orzalli and Kagan, 2017). The cGAS‒STING pathway and apoptosis have a complicated connection, including cGAS‒STING-mediated apoptosis and the activation of IRF3 in cells that are actively undergoing cell-intrinsic apoptosis (summarized in Figure 2). On the other hand, the caspase family is thought to inhibit the signaling pathway and cytokine synthesis during apoptosis, thereby avoiding excessive cytokine production.

In summary, our understanding of the biological functions of the caspase family and the cGAS‒STING signaling pathway is still in its early stages. An in-depth exploration of the interplay between the caspase family and the cGAS‒STING signaling pathway is needed, which will provide new insights into their innate immunity regulation, allowing us to use the cGAS‒STING signaling pathway in antiviral, anticancer, and autoimmune disease treatments. Despite the related researches having elaborated the mechanism behind this, outstanding questions remain to be answered. For example, the discovery of suppression of IFN-I signaling by caspases makes it difficult to explain the following phenomena: driving apoptotic caspases through viral infections is beneficial for the host to deprive the viral replication niche, which prevents replication of intracellular pathogens by elimination of infected cells by activating ‘suicidal’ cell death. In contrast, apoptotic caspase-mediated attenuation of the IFN-I-producing pathway allows the virus expansion. Presumably, there may be spatiotemporal regulatory mechanisms of apoptosis and IFN-I induction. Alternatively, there may be two functionally different cells: one preferentially induces apoptosis, and the other preferentially produces IFN-I.

It is necessary to ensure the elimination of pathogenic microorganisms and cancer cells by the cGAS‒STING signal pathway, at the same time, to prevent the excessive secretion of cytokines caused by its over-activation, which is the direct cause of the inflammatory factor storm. Therefore, timely and accurate response to different regulations of functional caspases is important to maintain the innate immunity mediated by cGAS‒STING.
